# Unusual suspects: Glial cells in fertility regulation and their suspected role in polycystic ovary syndrome

**DOI:** 10.1111/jne.13136

**Published:** 2022-04-20

**Authors:** Elodie Desroziers

**Affiliations:** ^1^ Department of Physiology, Centre for Neuroendocrinology University of Otago Dunedin New Zealand; ^2^ Sorbonne Université, CNRS, INSERM, Neuroscience Paris Seine – Institut de Biologie Paris Seine, Neuroplasticity of Reproductive Behaviours Team Paris France

**Keywords:** astrocyte, glia, GnRH, PCOS, tanycyte, microglia, fertility

## Abstract

Gonadotropin‐releasing‐hormone (GnRH) neurons sitting within the hypothalamus control the production of gametes and sex steroids by the gonads, therefore ensuring survival of species. As orchestrators of reproductive function, GnRH neurons integrate information from external and internal cues. This occurs through an extensively studied neuronal network known as the “GnRH neuronal network.” However, the brain is not simply composed of neurons. Evidence suggests a role for glial cells in controlling GnRH neuron activity, secretion and fertility outcomes, although numerous questions remain. Glial cells have historically been seen as support cells for neurons. This idea has been challenged by the discovery that some neurological diseases originate from glial dysfunction. The prevalence of infertility disorders is increasing worldwide, with one in four couples being affected; therefore, it remains essential to understand the mechanisms by which the brain controls fertility. The “GnRH glial network” could be a major player in infertility disorders and represent a potential therapeutic target. In polycystic ovary syndrome (PCOS), the most common infertility disorder of reproductive aged women worldwide, the brain is considered a prime suspect. Recent studies have demonstrated pathological neuronal wiring of the “GnRH neuronal network” in PCOS‐like animal models. However, the role of the “GnRH glial network” remains to be elucidated. In this review, I aim to propose glial cells as unusual suspects in infertility disorders such as PCOS. In the first part, I state our current knowledge about the role of glia in the regulation of GnRH neurons and fertility. In the second part, based on our recent findings, I discuss how glial cells could be implicated in PCOS pathology.

## Graphical abstract

Polycystic Ovary syndrome (PCOS) is the most common endocrine and anovulatory disorder in women of reproductive age worldwide. Although PCOS is commonly considered an ovarian disorder, human and animal‐based evidence demonstrate a miscommunication between the brain and the ovary. Fertility is well‐known to be controlled by a neuronal network in the brain. However, the brain is not only composed of neurons. In this review, I propose glial cells as major players in the establishment and maintenance of PCOS pathophysiology.

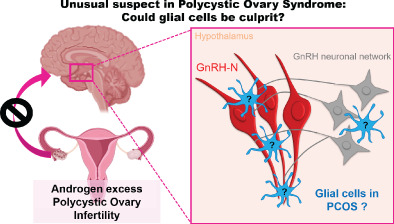

## SUMMARY FOR NON‐SPECIALIST

Polycystic ovary syndrome (PCOS) is the most common infertility disorder, affecting 5%–20% of reproductive aged women worldwide. Although PCOS is commonly considered an ovarian disorder, recent animal‐based evidence presents the brain as a prime suspect in PCOS pathology. Fertility is well‐known to be controlled by a neuronal network in the brain. However, the brain is not simply composed of neurons. Traditionally, glial cells have been regarded as passive contributors to brain function. Recent findings demonstrate that glial cell dysfunction contributes to brain disorders such as neuropsychiatric and neurological disorders. Despite some studies investigating the role of glial cells in fertility onset and regulation, the role of glia in fertility disorders such as PCOS remains underappreciated. In this review, I propose glial cells as major players in the establishment and maintenance of PCOS pathophysiology. To this end, I first state our current knowledge about the role of glial cells in fertility regulation and then discuss new evidence supporting glial cells as potential contributors to PCOS. To finish, I discuss future research avenues.

## 1 INTRODUCTION

The hypothalamic‐pituitary‐gonadal (HPG) axis regulates the timely production of gametes by male and female gonads essential to the survival of all mammalian species. The hypothalamus, based in the ventral part of the forebrain, is a highly conserved brain structure that integrates information from external and internal cues to orchestrate reproductive outcomes. Specifically, a subset of neurons located in the ventral part of the forebrain, the gonadotropin‐releasing‐hormone (GnRH) neurons, secrete the neurohormone GnRH. This neurohormone triggers the secretion of gonadotropins by the pituitary that then act on the gonads to produce sex steroids and gametes. Sex steroids feedback to GnRH neurons indirectly via a network of neurons located throughout the hypothalamus referred to as the “GnRH neuronal network.”^1^, ^2^ However, the brain is not simply composed of neurons.

Glial cells compose at least half of the brain depending on the region analysed. The cortex has a glia‐to‐neuron ratio (GNR) of 1:1; however, the brainstem, diencephalon and striatum have a GNR closer to 10:1.^3^, ^4^ Despite this, their functional role in the central control of fertility remains underappreciated. The classical view of glial cells in the brain is as mere supportive cells for neurons. The latest technological advances in the neuroscience field have led to a wealth of experimental studies demonstrating the functional roles of glial cells in shaping and regulating brain function during development, adulthood and aging.^5^, ^6^ In addition, recent studies have revealed that numerous pathologies of the nervous systems target glia.^5^, ^6^ Concerning the role of glia in the neuroendocrine regulation of fertility, pioneering studies in the early 1990s highlighted the role of glial cells on reproductive function not only through neuroanatomical studies of the relationship between GnRH neurons and glia, but also through investigation of the role of specific molecules known to be implicated in neuroglial communication.^7^ These findings promoted the role of the “GnRH glial network” in sexual maturation throughout postnatal development, as well as in relaying internal and external cues to the GnRH neurons to regulate the timely secretion of GnRH in females during puberty and adulthood. However, to date, numerous questions still remain regarding the specific role of different glial cell types in the control of GnRH neuron activity, GnRH secretion and fertility outcomes. Are all glial cell types associated with GnRH neurons? How are they regulating GnRH neuron activity and secretion? Could they play key roles in infertility disorders such as PCOS?

Outside the scope of fundamental knowledge on how the “GnRH glial network” regulates fertility, investigating the role of glial cells in infertility disorders could lead to the discovery of new targets and treatments in this world of increasing infertility disorders with one in four couples worldwide affected by infertility.^8^ Among women with infertility, polycystic ovary syndrome (PCOS) is the most common form of anovulatory infertility worldwide, currently affecting 5%–20% of women of reproductive age.^9^ As the name indicates, PCOS has long been seen primarily as an ovarian disorder. However, in PCOS patients, elevated luteinising hormone (LH) pulse frequency associated with impaired sex steroid feedback has led to the hypothesis that the communication between the brain and ovaries is impaired.^10^, ^11^ Subsequently, evidence from animal‐based models of PCOS has highlighted the role of the brain in this disorder and, more particularly, the role of the GnRH neuronal network.^12^, ^13^ However, a major neurocentric view of this pathophysiology has contributed to a lack of studies on the role of glial cells in this infertility disorder.

In this review, I aim to propose glial cells as unusual suspects in neuroendocrine infertility disorders such as PCOS. Leading to this idea, the first part of the review briefly develops the mechanism by which the brain regulates fertility and describes the current state of knowledge on the role of different glial cell types in fertility maturation and regulation with a focus on GnRH neuron function. In the second part of this review, I summarise how the communication between the brain and the ovaries is impaired in PCOS and discuss the potential role of some glial cell types in the establishment and maintenance of PCOS pathophysiology.

## 2 DO GLIAL CELLS PLAY A ROLE IN FERTILITY REGULATION?

**FIGURE 1:**
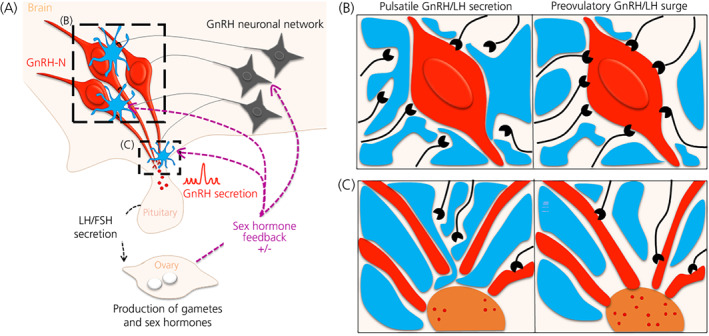
Role of glial cells in fertility regulation. (A) Schematic of the hypothalamic‐pituitary‐gonadal (HPG) axis: gonadotropin‐releasing hormone (GnRH) neurons (red) secrete GnRH within the portal blood to stimulate gonadotropin secretion by the pituitary. Then, luteinising hormone (LH) and follicle‐stimulating hormone (FSH) target the ovaries to trigger the production of gametes and sex steroid hormones. These sex hormones then feedback to the GnRH neurons via the “GnRH neuronal network.” Glial cells (blue) are observed in the vicinity of the GnRH neurons and nerve terminals and play a role in the regulation of GnRH secretion. (B) Role of glial cells around the GnRH soma: during the basal pulsatile GnRH secretion phase, glial cells (blue) enwrap GnRH neurons (red), blocking inputs (black); however, preceding the preovulatory GnRH surge, glial processes allow the GnRH neuronal networks inputs to connect to GnRH neurons, therefore triggering a GnRH surge. (C) Role of glial cells in the vicinity of GnRH nerve terminals: during the basal pulsatile GnRH secretion phase, glial cells (blue), mostly tanycyte end‐feets, enwrap GnRH nerve terminals (red) to block their access to portal blood capillaries (brown); however, at the time of the preovulatory surge, tanycyte end‐feets retract, allowing GnRH nerve terminals to access the portal blood capillaries, leading to high secretion of GnRH (i.e., the preovulatory surge). GnRH‐N, gonadotropin‐releasing hormone neurons

Fertility is controlled by a small population of brain cells known as GnRH neurons (Figure 1A). GnRH neurons secrete pulses of GnRH peptide into the pituitary portal system to drive the pulsatile release of LH and follicle‐stimulating hormone (FSH) from the pituitary gland. LH and FSH then act on the gonads to stimulate the production of gametes and sex steroid hormones in a sexually dimorphic manner. In females, once per reproductive cycle, GnRH secretion is increased in a surge pattern that triggers a preovulatory LH surge leading to ovulation. Circulating concentrations of sex steroid hormones are then conveyed to GnRH neurons; however, GnRH neurons do not express the sex steroids receptors that are essential for the feedback regulation of the HPG axis.^1^, ^2^ Positive and negative sex steroid hormone feedback has been shown to occur in part through a synaptically‐connected neuronal network commonly referred to as the ‘GnRH neuronal network’.^1^, ^2^ In addition, evidence supports an important role for glial cells in the relay of sex steroid feedback onto GnRH neurons. Sex hormones influence glial cell morphology, number and function throughout life in different brain regions.^14^ However, whether or not glial cells express sex steroid receptors depends on the glial cell type, the brain region and the physiological or pathological state of the animal studied.^14^, ^15^ In addition, glial cells have been shown to change their morphology and their secretome depending on external (light/season) and other internal (energy balance) cues, as well as to interact/communicate with GnRH neurons, therefore positioning them as important modulators of the central regulation of the HPG axis (Figure 1A).

### 2.1 Glial cells within the brain

Glial cells consist of radial glia, microglia, astrocytes, tanycytes and oligodendrocytes.^5^ The radial glia are the progenitors of the central nervous cells generating astrocytes and neurons within specialised neurogenic zones.^5^ Astrocytes, the star‐shaped cells of the brain, originate from the same neuroepithelial progenitors as neurons and share similar functions, such as expressing G‐protein coupled receptors (neuropeptides, neurotransmitter and hormones receptors)^15^, ^16^ and signalling molecules, capable of calcium signalling and releasing gliotransmitters.^5^, ^15^, ^17^ Astrocytes are mostly known to regulate neuronal activity through the concept of a tripartite synapse, such that they can modulate glutamate release and uptake within the synaptic cleft, therefore modulating synaptic transmission.^5^, ^18^ In addition, they have been shown to play a major role in remodelling neuronal circuitry during development and adulthood, being implicated in many relevant aspects of neuronal function, such as neuronal trophic support, control of ion homeostasis, neuronal survival and differentiation, neuronal guidance, neurite outgrowth, and synaptic efficacy.^5^, ^15^, ^17^ Tanycytes are a specialised type of ependymocytes lining the ventral part of the third ventricle. Tanycytes are currently classified into four subtypes: α1, α2, β1 and β2, depending on their dorsoventral locations within the third ventricular wall. However, with the advances in technology, new evidence from structural, functional and transcriptomic studies calls for a novel classification closer to their specific functions.^19^ Microglia are the resident immune cells of the brain. They originate outside the brain and populate the brain during early embryonic development.^20^ Microglia were first investigated with respect to their role as resident immune cells of the adult brain that phagocytose cell debris and recruit macrophages from the periphery during episodes of infections or brain insults such as stroke and ischemia, or in neurodegenerative diseases.^20^ However, it is now well established that microglia have a myriad of other functions that shape and maintain homeostasis in the brain. Microglia are now known for their active role in regulating neuronal wiring throughout development: from the induction of neuronal spine formation to their role in pruning and refining synapses during early development, contributing to the maturation of neuronal circuitries.^20^ In the hypothalamus, VanRyzin et al.^21^ showed that microglia are regulated by sex steroids and play a role in masculinising the rodent brain. Oligodendrocytes are the “Schwann cells” of the central nervous system producing myelin sheath for neurons of the central nervous system.^5^ Myelin forms an insulating layer around nerve terminals and is implicated in homeostasis and speeding‐up the electrical impulses for an efficient communication between central nervous system neurons and the peripheral nervous system.

### 2.2 From neuroanatomical evidence to a suggested functional role in fertility regulation

Microglia and oligodendrocytes are the forgotten glial cells when it comes to the GnRH neurons and fertility regulation. For microglia, to date, only two publications have shown microglia association with GnRH soma.^22^, ^23^ The first paper is a qualitative study observing microglia in association with GnRH neuron soma of adult female rats.^23^ The second paper is our recent study highlighting that, in addition to the presence of microglia in the vicinity of GnRH neurons, their filopodia ensheath GnRH neurons during postnatal development.^22^ Concerning oligodendrocytes, the recently published study by Pellegrino et al.^24^ showed, for the first time by immunohistochemistry, the presence of oligodendrocytes in the vicinity of GnRH neurons cell bodies during juvenile development in the female rat brain. Despite showing a physical association of GnRH cell bodies with microglia and oligodendrocytes, the role of these glial cell types in GnRH neuron activity, secretion and fertility regulation remains to be fully determined.

By contrast, astrocytes and tanycytes are well documented glial cell types in terms of proximity to GnRH neuron soma and terminals. They are also the most extensively studied for their role in regulating GnRH neuron activity, secretion and reproductive outcomes. Evidence supporting the physical and chemical interactions between GnRH neurons and these two glial cell types is discussed below.

#### 2.2.1 Glial plasticity in the vicinity of GnRH neurons

Coverage of GnRH neuron cell bodies by glial cells, identified by glial fibrillary acidic protein (GFAP), is dependent on developmental age^25^, ^26^ and hormonal status in different species.^27^, ^28^ In adult female monkeys, a comparison of the GFAP‐immunoreactive positive (GFAP‐IR^+^) coverage at different stages of the menstrual cycle or by artificial treatment with sex steroids (ovariectomy with or without estradiol replacement), suggests that GFAP‐IR^+^ cells are influenced by sex steroid levels and can change their morphology accordingly.^28^ Interestingly, in their study, Witkin et al.^28^ observed that the degree of ensheathment of other non‐identified neurons within the preoptic area (POA) does not vary systematically after hormonal manipulations compared to the degree of ensheathement of GnRH neurons, suggesting that these hormone‐dependent changes in glial ensheathment are specific to GnRH neurons. Other in vitro and in vivo studies in rodents also showed that glial cells are sensitive to sex steroids in the vicinity of GnRH neurons in the hypothalamus,^29^, ^30^, ^31^ as well as other parts of the brain.^14^ Together, these results led to the concept that glial cells could relay sex steroid feedback to GnRH neurons. In addition, another morphometric study in female rats observed a diurnal plasticity around the time of the preovulatory surge, suggesting that astrocytes also integrate and relay external cues to GnRH neurons.^27^ These morphological changes in the rostral POA could be interpreted as a sex‐steroid dependent switch in astrocytic ensheathment of the GnRH neuron cell bodies facilitating the interaction between excitatory inputs and the GnRH neuron soma at the time of pubertal onset and preceding the preovulatory surge (Figure 1B). These changes in ensheathment could either: (1) physically allow neuronal afferent inputs to communicate with GnRH neuron perikarya to relay information from peripheral cues and/or (2) relate to a shift of astrocytic processes toward active dendritic spines to regulate synapse homeostasis. This last mechanism has been demonstrated in mouse hippocampal cultures where astrocytes prefer association with large mushroom‐like postsynaptic dendritic spines.^32^

At the level of GnRH neurons nerve terminals, GFAP‐IR^+^ cells, also known as astrocytes, and vimentin‐immunoreactive positive cells, a marker of tanycytes, have been observed. It is well‐known that GnRH neuron nerve terminals make physical contact with the pericapillary space of the median eminence and that this contact increases during the preovulatory phase (GnRH/LH surge leading to ovulation) compared to the luteal phase (basal GnRH/LH pulsatile secretion). Through a series of elegant studies, the research group led by Vincent Prevot showed that tanycytes are essential components of this structural plasticity in rats,^33^ as well as in humans.^34^, ^35^ During the luteal phase, tanycytic end‐feet have been shown to physically restrict access of the GnRH nerve terminals to the portal blood vessels (Figure 1C). Then, at the time of the preovulatory surge, tanycytes physically allow GnRH nerve terminals to contact the portal blood vessels by retraction of their end‐feet (Figure 1C). This structural plasticity has also been shown to be dependent upon estrogen signalling.^36^ Therefore, tanycytes act as a physical barrier allowing or restricting GnRH neuron nerve terminals access to the portal blood and are an essential component of the regulation of GnRH secretion dependent on sex steroids signalling. Numerous studies have subsequently shed light onto the mechanisms by which this structural remodelling happens, involving complex communication between glial cells and GnRH neuron soma and nerve terminals.^7^

#### 2.2.2 GnRH–glial communication

**FIGURE 2:**
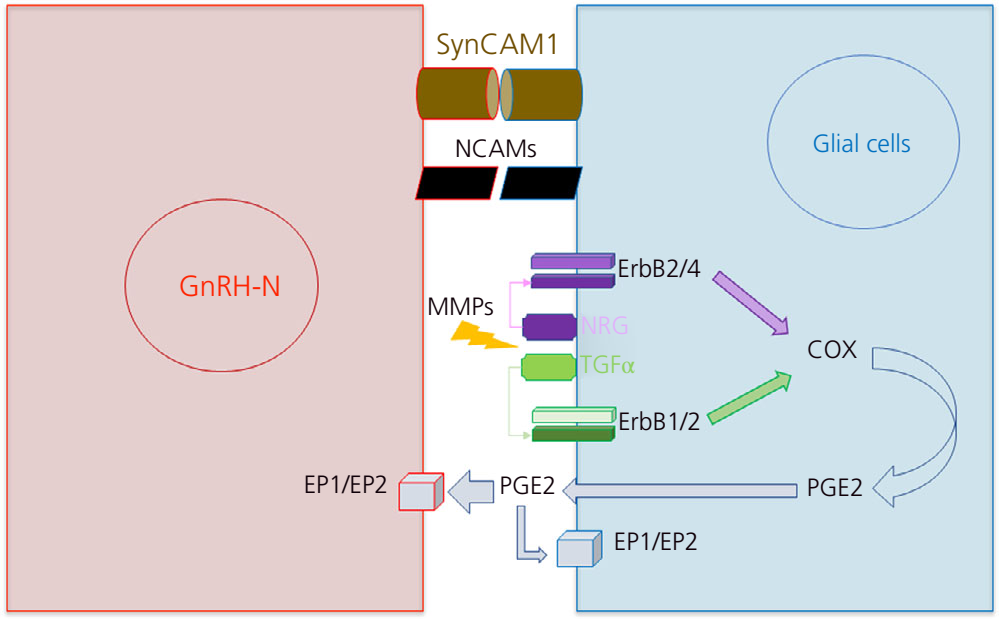
Communication between gonadotropin‐releasing hormone (GnRH) neurons and glial cells. Numerous signaling molecules are involved in the communication between glial cells and GnRH neurons (GnRH‐N). The following molecules have been shown to contribute to the regulation of GnRH secretion by astrocytes and tanycytes: the adhesion molecules synaptic cell adhesion molecule 1 (SynCAM1) and neural cell adhesion molecule (NCAM), as well as some glial factors and their receptors such as prostaglandin E2 (PDGE2), transforming growth factor α (TGFα) and neuregulin (NRG), and their receptors, respectively, EP1/2 and ErbBs (ErbB1/2 or ErbB2/4). COX2, cyclooxygenase 2; MMP, matrix metalloproteinase

First of all, the role of adhesion molecules in the GnRH neuron–glial communication has been well‐established over the years in different species.^37^, ^38^, ^39^, ^40^, ^41^, ^42^, ^43^, ^44^, ^45^ Two major adhesion molecules have been widely studied and shown to regulate GnRH secretion and fertility: synaptic cell adhesion molecule type 1 (SynCAM1) and the polysialylated form of the neural cell adhesion molecule (PSA‐NCAM) (Figure 2). Both are expressed by GnRH neurons and hypothalamic astrocytes in rodents^38^, ^41^ and in sheep,^44^, ^45^ as well as in the ex vivo GnRH neuron embryonic nasal explant mouse model.^44^, ^46^ PSA is known to block the homophilic interaction of NCAMs from two interacting cells, therefore blocking the communication between these cells.^44^ Removal of the PSA form of the NCAMs from cell surface by treatment with the enzyme endoneuraminidase N (EndoN) decreases GnRH secretion in the ex vivo GnRH neurons from embryonic nasal explants (I. Franceschini & E. Desroziers, unpublished data,^44^) and changes the morphology of GnV3 (immortalised GnRH neurons) cells in vitro.^37^ Interestingly, treating in vitro hypothalamic astrocytes with EndoN blocks the morphological changes observed following estradiol treatment, suggesting that PSA‐NCAM is required for the estradiol‐dependent morphological changes of hypothalamic astrocytes.^30^ In addition, treating adult female rats in vivo with EndoN within the mediobasal hypothalamus decreases GnRH‐immunoreactivity at the same time as increasing GFAP‐immunoreactivity accompanied by a disrupted estrous cycle.^37^ Altogether, these findings argue that communication between glial cells and GnRH neurons through NCAMs is important in the regulation of GnRH expression and secretion, as well as reproductive outcomes. Similarly, the deletion of the SynCAM1 specifically in GFAP‐expressing cells delays pubertal onset and impairs reproductive function in mice.^40^ Another type of neuroglial signalling has been observed in the median eminence, where a complex juxtacrine communication between GnRH neurons–tanycytes and tanycytes–endothelial cells of the blood–brain barrier is required for the integration of hormonal changes throughout the estrous cycle leading to the observed structural remodelling.^7^, ^19^ GnRH neuron and tanycyte communication occurs not only through adhesion molecules, but also through semaphorin‐7A and its receptor plexinC1 on GnRH neurons and β1‐integrin on tanycyte end‐feet.^43^ In conjunction, tanycyte–endothelial communication occurs through nitric oxide and growth factors dependent upon estrogen signalling.^36^, ^47^

Second, hypothalamic astrocytes and tanycytes have been shown to produce several growth factors such as transforming growth factor (TGF)β, insulin‐like growth factor‐1 and epidermal growth factor‐like peptides (TGFα and neuregulin1) during puberty and adulthood, which then regulate GnRH neuron activity and secretion (Figure 2).^7^ The first evidence for this arose from studies in which applications of TGFα to ex vivo median eminence explants was found to increase GnRH release into the culture medium.^48^ Thereafter, Ojeda et al.^49^ showed, using in vitro studies with immortalised GnRH neurons (GT1‐7 and GnV3 cells), as well as in vivo loss of function within GFAP expressing cells, a stimulatory effect of glial derived growth factors (TGFα and β, neuregulin) on GnRH release. This occurs either by direct action on their receptors (ErbB1, ErbB4&2) expressed by GnRH neurons or through indirect actions onto neighbouring astroglial cells producing prostaglandins (PGE2), which in turn act on GnRH neurons expressing the PGE2 receptor.^7^, ^49^ More precisely, astroglial PGE2 controls GnRH neuronal firing through the EP2 receptor at the level of the soma,^50^ whereas PGE2 acts through the EP1 receptor at the level of the GnRH nerve terminals to regulate GnRH secretion.^51^ Recently, it has been suggested that GnRH neurons are also able to recruit astrocytes during the juvenile period in rodents through prostaglandin signalling.^24^ Finally, another type of cell–cell communication has also been suggested to modulate GnRH secretion. Astrocyte–astrocyte communication through connexin 43, a gap junction expressed predominantly in astrocytes, has been shown to regulate GnRH secretion in ex vivo GnRH neurons from embryonic mouse nasal explants in culture.^52^ Indeed, treatment with a pharmacological blocker of connexin 43 led to a decrease in GnRH activity and pulsatile secretion.^52^

These mechanisms of action by which glial cells modulate GnRH activity and secretion have been highlighted only for astrocytes and tanycytes in the vicinity of GnRH neurons soma and nerve terminals. In brief, these results can be interpreted as a modulation of GnRH–glial signalling (Figure 2) leading to structural changes dependent upon the physiological status of the animal (Figure 1). These structural changes, in turn, may decrease glial cell ensheathment around GnRH cell bodies and nerve terminals, leading to increased access of other neuronal inputs onto GnRH neuron soma and dendrites, as well as an increased accessibility of the GnRH neuron nerve terminals to capillary blood, in times of high GnRH activity and secretion such as pubertal onset and the preovulatory surge (Figure 1).

**FIGURE 3:**
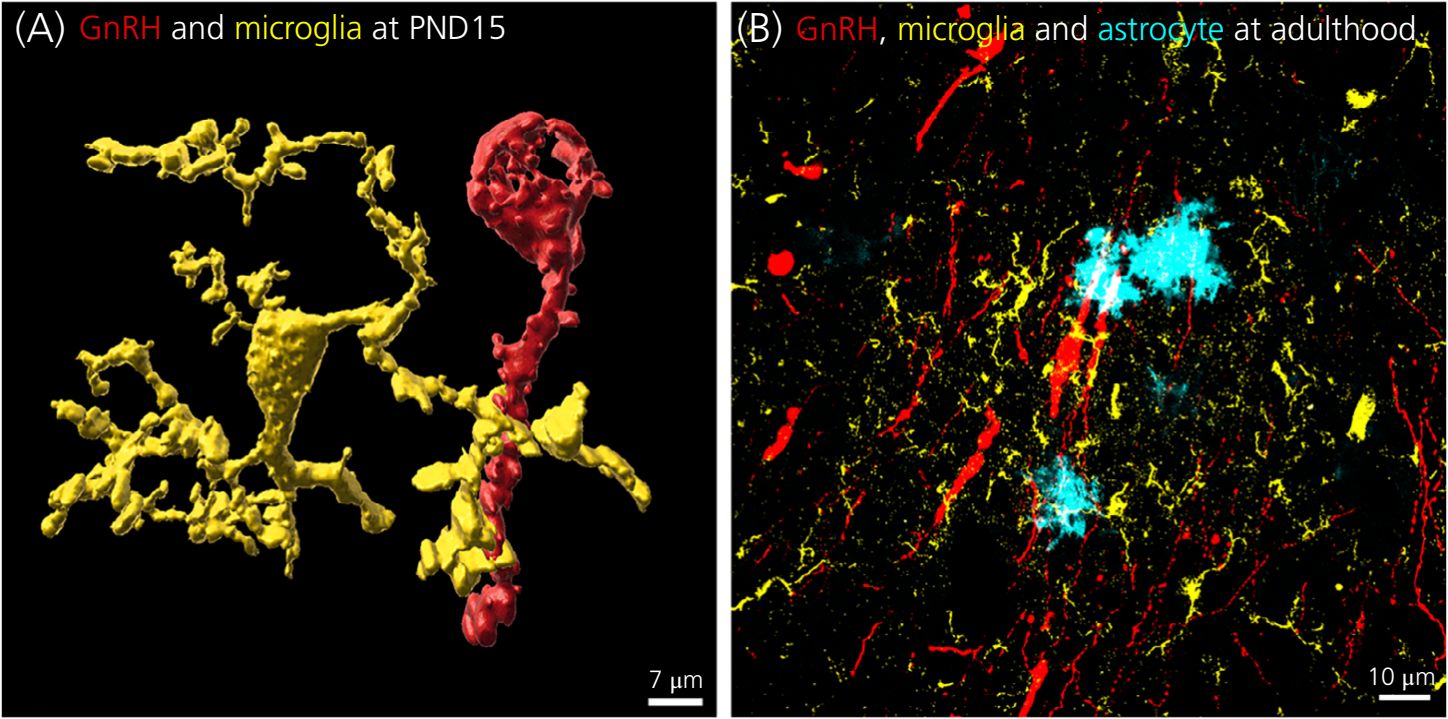
Physical interaction between gonadotropin‐releasing hormone (GnRH) neurons, microglia and astrocytes in the mouse brain during development (A) and adulthood (B). (A) Microglia (CX1CR3‐GFP, yellow) are in close association with the soma and dendrites of a GnRH neuron (GnRH‐immunoreactivity, red) within the rostral preoptic area at postnatal day 15 in mice. In this 3D reconstruction from confocal micrographs, one of the microglia filopodia (yellow) enwraps the GnRH dendrite (red) (images adapted from Sati et al.^22^). (B) Microglia (IBA‐1 immunoreactivity, yellow) are also found in the vicinity of GnRH neurons soma and dendrites (GnRH‐immunoreactivity, red) in the rostral preoptic area at adulthood. In this representative single focal plan (*z* = …) taken by a confocal microscope, we observe that microglia (yellow) are not only in the vicinity of GnRH neurons, but also in close association. Interestingly, we can observe that astrocyte (Aldh1L1‐cre/dTomato, cyan) are also in close association with both GnRH neurons (GnRH‐immunoreactivity, red) and microglia (IBA‐1‐immunoreactivity, yellow) (E. Desroziers, unpublished results). PND, postnatal day

New advances in technological tools are refreshing the ability to question the functional role of glial cells in the regulation of fertility. For example, a recent elegant study used chemogenetic tools, also known as DREADDs (i.e., Designer Receptors Exclusively Activated by Designer Drugs), to activate GFAP‐expressing cells in mice within the vicinity of GnRH neurons. This study showed that selectively activating GFAP‐expressing cells in the vicinity of GnRH neuron soma led to an increase in LH secretion in male mice.^53^ Noteworthy, when trying to also elucidate the role of GFAP‐expressing cells on GnRH/LH secretion in females, Vanacker et al.^53^ encountered an unexpected sex difference in the expression of DREADD, with a higher percentage of the cells transfected with DREADD in females showing a neuronal phenotype compared to males. In addition, Vanacker et al.^53^ were unable to elucidate LH secretion in females following activation of GFAP‐expressing cells in the vicinity of GnRH neurons soma. These observations not only suggest that GFAP‐expressing cells show phenotypic and functional differences between male and female brains in the hypothalamus, but also highlight GFAP as an imperfect way of targeting astrocytes. Indeed, numerous previous studies have observed that transgene expression driven by the human or mouse GFAP promoter does not occur in all astrocytes, and is also often detected in neurons depending on the brain region and physiological states of the animal studied.^54^, ^55^ New genetic lines based on the astrocyte‐specific enzyme aldehyde dehydrogenase family 1 member L1 (Aldh1L1) appear to achieve a more complete and specific astrocyte targeting.^55^ Our preliminary results show that Aldh1L1‐cre/dTomato cells (Figure 3, cyan) are also found in close apposition with GnRH neurons (Figure 3, red) in adult male mouse brains (E. Desroziers, unpublished data). Analysis of the association between GnRH neurons and Aldh1L1‐cre/dTomato cells in male and female mice over the estrous cycle will determine whether Aldh1L1‐cre mice are a better tool for investigating the functional role of astrocytes in female fertility regulation.

Taken together, these important studies have highlighted a role for glial cells in GnRH neuron regulation and female reproductive outcomes, providing a solid background with which to continue the exploration of the functional role of glial cells in fertility and infertility disorders.

## 3 DO GLIAL CELLS PLAY A ROLE IN THE NEUROENDOCRINE PATHOLOGY OF PCOS?

### 3.1 PCOS: A miscommunication between the brain and the ovaries

**FIGURE 4:**
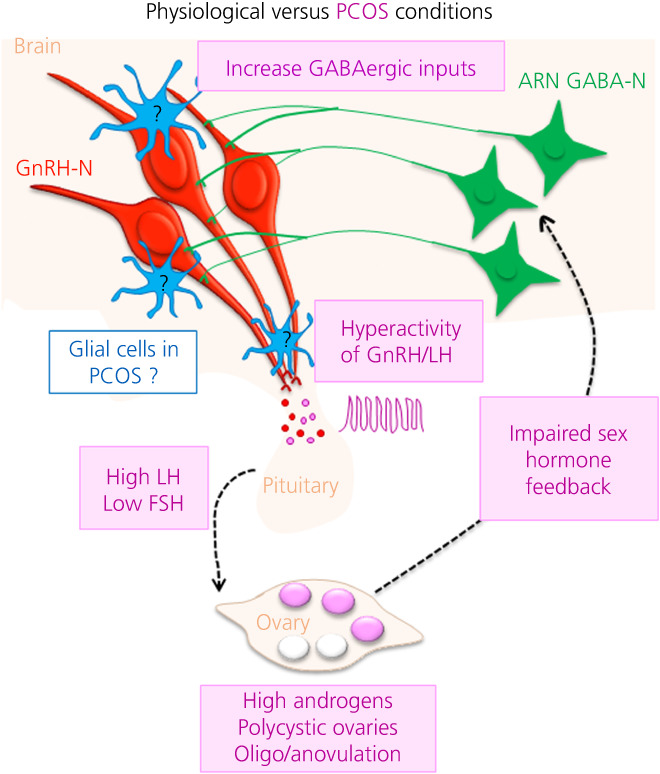
Unravelling the role of glial cells in polycystic ovary syndrome (PCOS)‐pathological regulation of fertility. Schematic illustrating current knowledge on the role of the brain in the regulation of the hypothalamic‐pituitary‐gonadal (HPG) axis in PCOS. In PCOS, an increase pulsatile secretion of luteinising hormone (LH) and lower secretion of follicle‐stimulating hormone (FSH) has been observed, mirroring a hyperactivity of gonadotropin releasing hormone neurons (GnRH‐N) (red), leading to the three cardinal features of PCOS: high circulating level of androgens, polycystic ovaries and oligo/anovulation. Impaired sex hormone feedback has also been observed in patients with PCOS. Animal‐based models have allowed to investigate the role of the brain in this disruption of the HPG axis. It has been observed that GABA neurons coming from the arcuate nucleus (ARN GABA‐N, green) are less sensitive to at least one sex hormone and send more projections to the GnRH neurons, leading to the hypersecretion of GnRH/LH. We now wonder whether glial cells (blue) could play a major role in the PCOS pathophysiology. ARN GABA‐N, GABA‐N located in the arcuate nucleus

PCOS is the most common anovulatory infertility disorder, affecting between 5% and 20% of women of reproductive age worldwide.^9^ PCOS is characterised by the presence of at least two of three diagnostic criteria: elevated androgen hormones, menstrual dysfunction and multiple cyst‐like follicles in the ovary.^56^ In PCOS patients, LH pulse frequency, which mirrors GnRH neuron activity, is significantly increased.^57^ Clinical data have demonstrated impaired estrogen and progesterone feedback in PCOS patients.^10^, ^11^ This clinical presentation suggests the brain is a major culprit of PCOS pathology (Figure 4).

Animal‐based models of PCOS have helped us to understand the underlying mechanism of this impaired sex steroid feedback.^12^, ^13^ Specifically, several studies have demonstrated that GnRH neuron activity or GnRH/LH secretion is increased in animal models of PCOS that recapitulate the three hallmarks of PCOS: the prenatally androgenised (PNA) and the prenatally Anti‐Mullerian hormone treated (PAMH) models of PCOS.^58^, ^59^, ^60^, ^61^, ^62^, ^63^ In the PNA and PAMH mice, a hyperactivity of GnRH neuron together with an increased number of dendritic spines on GnRH neuron perikarya have been observed.^59^, ^60^, ^62^ Moreover, this is associated with an increase in excitatory GABAergic neurotransmission and putative innervation to GnRH neurons^59^, ^61^, ^62^, ^63^ (Figure 4). Increased GABA afferent inputs onto GnRH neurons largely originate from GABA cell bodies located in the arcuate nucleus (ARN GABA) in the PNA mice^59^ (Figure 4). In addition, my previous work^64^ has used chemogenetic tools to chronically activate the ARN GABA aiming to determine whether activation of this population was sufficient to evoke PCOS‐like features. It was determined that chronic activation of ARN GABA neurons in healthy female mice disrupted estrous cyclicity and ovarian morphology and elevated testosterone, supporting a functional role for ARN GABA neurons in the development of PCOS.^64^ Finally, a role for elevated GABA in PCOS pathology is also supported by data obtained in women showing higher levels of GABA detected in the cerebrospinal fluid of PCOS patients compared to controls.^65^

Other work in the PNA mouse model of PCOS has found that increased excitatory GABA‐N inputs onto GnRH neurons are established prior to the disease onset.^66^, ^67^ These findings suggest that aberrant neuronal wiring of GABAergic inputs play a role in PCOS development. However, the mechanisms by which this impaired neuronal wiring is established during development remain unclear. When considering the potential mechanisms, a role for glial cells in shaping and rewiring the “GnRH neuronal network” cannot be neglected.

### 3.2 Unusual suspect: Potential role of glial cells in PCOS

As previously detailed, glial cells are now recognised as major players in brain development and brain function, as well as for playing a major role in disease onset and progression.^5^, ^6^ Here, my working hypothesis is that, in PCOS, not only the “GnRH neuronal network”, but also the “GnRH glial network” can be disrupted (Figure 4). Our recent work highlighted the role of one glial cell type in the development of aberrant neuronal GABA wiring during development (Figure 4), therefore opening a novel avenue to investigate the role of glial cells in the etiology and maintenance of PCOS pathophysiology.

#### 3.2.1 A role for microglia in shaping the PCOS brain

**FIGURE 5:**
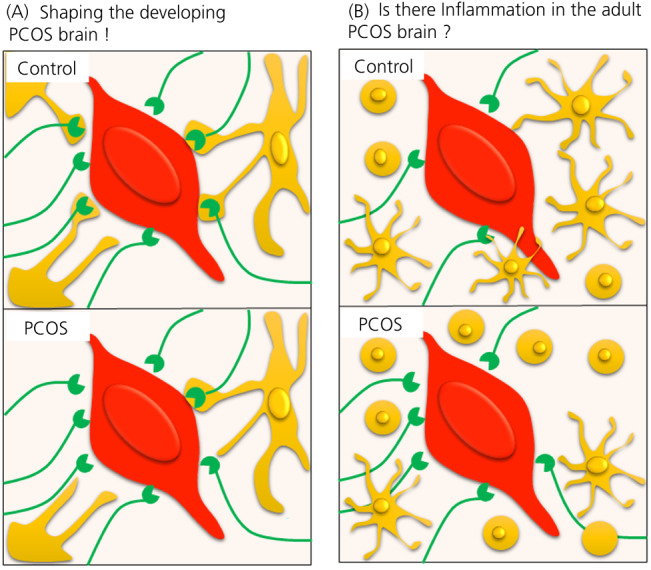
Role of microglia in polycystic ovary syndrome (PCOS) pathology. Schematic illustrating our recent findings on the role of microglia in PCOS. (A) Microglia are shaping the developing PCOS brain: recent evidence showed that microglia (yellow) engulf (i.e., eat) GABAergic inputs (green) in the vicinity of the soma of gonadotropin‐releasing hormone (GnRH) neurons (red); however, in the PCOS condition, microglia engulf/eat less GABAergic inputs, leading to the observed increase in GABAergic inputs at adulthood. (B) Is there inflammation in the adult PCOS brain? Our current results show a subtle increase of the inflammatory microglia phenotype (i.e., ameboid microglia) (yellow) in the vicinity of the soma of GnRH neurons (red), suggesting a potential inflammatory status in the prenatally‐androgenised mouse model of PCOS; however, further investigations are still needed to draw any conclusions

Microglia are well‐known to refine neuronal circuitry during development by inducing neuronal spine formation, as well as pruning and refining synapses, and contributing to the maturation of neuronal circuitry.^20^ A transient depletion of microglia during postnatal development (between postnatal day [P]8 and P28) is known to result in the retainment of excessive immature but functional synapses.^68^ Transient depletion of microglia around P11 has been shown to impair sex steroid‐mediated feedback, pubertal onset and oestrus cyclicity,^69^ suggesting that microglia are required for establishing normal endocrine function in adulthood. In a recent study, using immunohistochemistry, we compared microglia number and phenotype within two regions of the hypothalamus in the PNA mouse model of PCOS and health controls throughout development and in adulthood.^22^ We found a decrease in the total number of microglia in PNA mice at P25, before disease onset (Figure 5A) and in alignment with the observation of increased GABAergic inputs. This decrease was restricted to the thick microglia phenotype, which is known to be a phagocytic phenotype.^20^ Phagocytic function of microglia was then observed during the well‐characterised intense synapse pruning period that occurs between P7 and P15 in the mouse. Our findings suggested an impaired phagocytic activity specifically targeted to GABA inputs in the rostral POA with microglia pruning fewer GABAergic terminals at P15 in the PNA mouse model of PCOS (Figure 5A).^22^ These results are in accordance with a recent study using in vivo imaging to demonstrate that microglia interact with cortical GABAergic synapses, around P12‐P17, to phagocytose and thus refine GABAergic inputs.^70^ Therefore, these findings support an important role for microglia in the onset of prenatal androgen mediated PCOS‐like features.

#### 3.2.2 Do microglia play a role in adult PCOS pathology?

Microglia are the resident immune cells of the brain and therefore their major role in adulthood is to survey the brain parenchyma and trigger inflammatory responses after a pathogen's invasion or any insults within the brain.^20^ Microglia respond to inflammation by producing numerous factors such as pro‐ and anti‐inflammatory cytokines and prostaglandins,^20^ which have been shown to impair the central regulation of fertility in females.^71^ Clinical and pre‐clinical evidence suggests that PCOS may be associated with an inflammatory state. Elevated peripheral inflammatory markers including cytokines have been reported for PCOS patients from different populations.^72^, ^73^ Clinical studies have measured circulating pro‐ and anti‐inflammatory cytokines in PCOS patients, although reports have been contradictory for a number of these markers. For example, for the proinflammatory cytokines tumor necrosis factor α and interleukin‐6, some studies detected an increased level in the blood of PCOS patients, whereas others report no differences with the control group.^74^, ^75^, ^76^, ^77^ Therefore, there is an on‐going debate about whether or not PCOS is a chronic inflammatory disorder.^78^ Interestingly, reports from pre‐clinical studies have also assessed the inflammatory environment in a variety of rat models of PCOS and reported increased pro‐inflammatory factors.^79^, ^80^, ^81^ In the adult PNA mouse model of PCOS, we observed that microglia are largely unchanged apart from a very subtle increase in the ameboid phenotype that is associated with inflammation in the adult mice brain^22^ (Figure 5B). Thus, to date, whether this finding is associated with peripheral inflammation in the PNA mouse model of PCOS remains to be determined.

To conclude, our current results support a role for microglia in the etiology of PCOS and are the first to indicate a role for microglia in the brain in PCOS pathophysiology. However, numerous questions remain unanswered. Is the subtle increase in ameboid microglia in adulthood exacerbating PCOS symptoms or “simply” a response to peripheral inflammation? Are other glial cell types also implicated in PCOS origins and maintenance?

#### 3.2.3 What about the other type of glial cells?

To date, no studies have focused on the role of astrocytes, tanycytes or oligodendrocytes in PCOS pathology.

Given their role in regulating GnRH activity and secretion, I propose some hypotheses on how astrocytes and tanycytes could be implicated in PCOS pathophysiology. First, regarding the role of astrocytes and tanycytes in PCOS pathology, it could be that the physical ensheathment of GnRH neurons by those glial cells is disrupted in PCOS, thus enhancing the ability for excitatory inputs to access GnRH neurons and/or increase GnRH terminal access to the portal blood capillaries, leading to hypersecretion of GnRH/LH. In addition, an impaired communication between astrocytes and/or tanycytes with GnRH neurons could also lead to disrupted fertility outcomes similar to that observed with the modulation of neuroglial communication through adhesion molecules, growth factors and prostaglandin signalling in the vicinity of GnRH neurons, which have been shown to lead to impaired pubertal onset and oestrous cyclicity (see earlier section on GnRH–glial communication). Interestingly, in line with this hypothesis, a recent large‐scale genome‐wide meta‐analysis of PCOS patients has identified genetic variants in the ERBB4 loci.^82^ As highlighted in the first part of this review, a deletion of ErbB4 in GFAP‐expressing cells drive delayed puberty onset, impaired sex steroid feedback and fertility outcomes.^50^, ^83^ Therefore, a miscommunication between GnRH and astrocytes through impaired ErbB signalling remains to be investigated in PCOS animal models.

Second, astrocytes could also play a role in PCOS pathology by acting on other neurons within the “GnRH neuronal network.” Kisspeptin (Kp) neurons, recognised as key regulators of GnRH/LH secretion,^84^ are one possible target. Interestingly, a recent body of work focused on the role of astrocytes in regulating the RP3V Kp neuronal population. More particularly, astrocytic production and diffusion of neuroprogesterone has been shown to activate progesterone signalling in Kp neurons in vitro.^85^, ^86^ However, the role of Kp in PCOS remains controversial. Despite findings of increased circulating kisspeptin in the blood of PCOS patients, the results from animal models of PCOS measuring the expression of the Kiss1 gene or kisspeptin immunoreactivity are inconsistent, observing either an increase, decrease or absence of change.^87^ Therefore, not only the role of Kp neurons, but also their potential regulation by astrocytes in PCOS remains to be investigated. A second possible target is the ARN GABA neurons. Excess ARN GABAergic inputs to GnRH neurons are observed in two mouse models of PCOS and in the prenatally androgenised sheep model of PCOS.^59^, ^62^, ^63^ My previous work found that chronic activation of ARN GABA neurons with chemogenetics leads to the development of some PCOS‐like features in otherwise healthy female mice.^64^ Given that astrocytes and microglia express GABA receptors and that their activities can be modulated by GABA,^88^, ^89^ it remains of interest to determine whether and how GABA signaling and glial cells intersect in the mechanisms underlying PCOS. Indeed, recent studies investigating the homeostatic control of feeding behaviours have identified bidirectional communication between agouti‐related peptide (AgRP) neurons and astrocytes in the ARN. More particularly, the AgRP neurons release GABA, which increases astrocytic coverage of AgRP neurons. Next, astrocytes release PGE2, which increases the AgRP neurons excitability through PGE2/EP2 signalling.^90^ Interestingly, microglia have also been shown to be activated by GABA in the hippocampus.^91^ Therefore, we might hypothesise that increased GABA neurotransmission in the vicinity of GnRH neurons that is associated with the development of PCOS features may also lead to astrocytic and/or microglia activation. Although microglia remain largely unaffected in the adult PNA mouse model of PCOS, with only a modest increase in activated microglia,^22^ it remains to be determined whether other glial cells are affected and whether this is downstream from GABA actions. Together, these findings pose a question. Could excess GABA inputs and neurotransmission to GnRH neurons associated with PCOS impact glial activity around GnRH neurons and contribute to the GnRH/LH hyperactivity in PCOS?

Third, microglia and astrocytes have been shown to bi‐directionally communicate together, which determines the functional state of each cell type and can lead to central nervous system disease when disrupted.^92^ Interestingly, by immunohistochemistry, we observed a close association between microglia and astrocytes in the vicinity of GnRH soma (Desroziers E, unpublished data) (Figure 3). This observation suggests a potential astrocyte–microglia crosstalk dysfunction that could play a role in PCOS pathophysiology.

Finally, as discussed in the first part of this review, the first study showing oligodendrocytes in close association with GnRH neurons has been published and has reported observing oligodendrocytes within the vicinity of GnRH soma in the brain of juvenile rats.^24^ Interestingly, it has been observed that women affected by multiple sclerosis (MS), a demyelinating disease, are also affected with reproductive dysfunction, such as by an increase in serum levels of LH and FSH, a decreased serum estrogen concentration, menstrual irregularities and infertility.^93^ This indicates that degeneration of the myelin sheath in the brain could impact neurons regulating fertility. Noteworthy, MS is also an autoimmune disorder where the immune system attacks the neurons and is responsible for the demyelination of the nerve terminals and, as such, it cannot be ruled out that the resident immune cells of the brain, microglia, could also be the culprit in the infertility observed in women with MS. Therefore, it would be of interest to deepen the investigation of the role of oligodendrocytes in GnRH function, fertility regulation and PCOS pathophysiology.

Altogether, astrocytes, tanycytes and oligodendrocytes represent potential contributors to PCOS pathophysiology. Whether those glial cells are targets or culprits of PCOS development and pathophysiology remains to be determined. Because the clinical presentation of PCOS highlights the existence of at least four phenotypes of PCOS, depending on the number of diagnostic features present and the presence of co‐morbidities, it will not be surprising if different types of glial cells and mechanisms are involved in distinct phenotypes.

## 4 CONCLUSIONS

Glial cells are now seen as major players in the development of the central nervous system and as regulators of adult neuronal function. Pioneers and elegant studies have led the way to studies of the role of glial cells in the regulation of GnRH activity, secretion and fertility. Those studies have demonstrated sex steroid‐dependent morphological changes of glial cells around GnRH neurons and identified signalling molecules implicated in the GnRH neuroglial communication under different physiological conditions. They have also shown that disruption of the GnRH–glial physical association or communication leads to reproductive dysfunction. Armed with this knowledge and the technological advances to target specific glial cell types coupled with the use of a viral‐vector to specifically modulate gene expression and cell activity, we can now dream of ways to refine our current knowledge about the role of glial cells in the regulation of fertility, as well as uncover new mechanisms by which each glial cell types could regulate fertility outcomes. In addition, these findings question the role of glial cells in infertility disorders such as PCOS. Our recent work has shown a role for microglia in the establishment of PCOS pathology, although other glial cell types could also be culprits. By investigating the role of glial cells in PCOS pathophysiology, we will increase our understanding of PCOS disease mechanisms and potentially uncover future avenues with respect to prevention and treatment.

## CONFLICTS OF INTEREST

The author declares that she has no conflicts of interests.

### PEER REVIEW

The peer review history for this article is available at https://publons.com/publon/10.1111/jne.13136.

## Data Availability

Data sharing is not applicable to this review because no new data were created or analysed.
